# Depression care trajectories and associations with subsequent depressive episode: a registry-based cohort study (The Norwegian GP-DEP study)

**DOI:** 10.1186/s12875-025-02825-x

**Published:** 2025-04-24

**Authors:** Sharline Riiser, Tone Smith-Sivertsen, Valborg Baste, Inger Haukenes, Øystein Hetlevik, Sabine Ruths

**Affiliations:** 1 Department of Global Public Health and Primary Care, University of Bergen, Bergen, Norway; 2 Research Unit for General Practice, NORCE Norwegian Research Centre, Bergen, Norway; 3 Division of Psychiatry, Haukeland University Hospital, Bergen, Norway; 4 National Centre for Emergency Primary Health Care, NORCE Norwegian Research Centre, Bergen, Norway

**Keywords:** Depression, Health care services research, Antidepressant medication, Talking therapy, General practice, Specialist care

## Abstract

**Background:**

Depression often has a recurrent course, but knowledge about the impact of treatment trajectories is scarce. We aimed to estimate treatment trajectories for patients with recurrent depression, and to explore associations between the trajectories and subsequent depressive episodes.

**Methods:**

Cohort study based on linked registry data, comprising all Norwegian residents ≥ 18 years with an (index) depressive episode in 2012 following previous episode(s) in 2008–2011. We generated multi-trajectories based on treatment during index episode including GP follow-up consultation(s), long consultation(s) and/or talking therapy (with GP), antidepressants, and contact(s) with specialist care. Generalized linear models were used to analyse associations between different treatment trajectories and subsequent depression within one year.

**Results:**

The study population consisted of 9 027 patients, mean age 44.6 years, 63.9% women. Five treatment trajectory groups were identified: “GP 1 month” (45.2% of the patients), “GP 6 months” (31.9%), “GP 12 months” (9.3%), “Antidepressants 12 months” (9.0%), and”Specialist 12 months” (4.6%). In group”GP 1 month” (reference), 25.1% had subsequent depression. While trajectory group “Antidepressants 12 months”, had similar likelihood of subsequent depression as the reference (Relative risk (RR) = 1.04, 95% confidence interval (CI) 0.91–1.18), the groups “GP 12 months” (RR = 1.43, CI 1.28–1.59), “Specialist 12 months” (RR = 1.26, CI 1.08–1.47) and “GP 6 months” (RR = 1.17, CI 1.07–1.26) had increased risk of subsequent depression.

**Conclusions:**

Our findings suggest that long-term antidepressant treatment of patients with recurrent depressive episodes may prevent subsequent depression episodes. However, this finding needs to be confirmed through studies that take into account the severity of depression.

**Supplementary Information:**

The online version contains supplementary material available at 10.1186/s12875-025-02825-x.

## Background

There has been a change in our understanding of depression, from an acute self-limiting disorder to a relapsing–remitting condition with a chronic course for many patients [1]. After their first depressive episode, almost half of patients experience relapse or recurrence, mostly within the first six months [2]. Risk factors for recurrence includes number of previous episodes, severity of depression and residual symptoms after treatment [3].

A substantial proportion of patients with depression are diagnosed and managed by a general practitioner (GP) [4]. International guidelines [5] recommend psychological and/or pharmacological interventions for acute depression [6], but despite adequate treatment, the risk of recurrence remains high [7]. It is therefore important to gain insight into whether different treatment courses can prevent recurrence. Systematic reviews and meta-analyses [8–11] have documented that long term treatment with antidepressants can reduce relapse rates of depression up to 70% compared to placebo. Although these studies show robust findings of the protective effect of antidepressants, the included trials were mainly conducted in secondary mental healthcare, with patients at high risk of relapse. Further, the trials were heterogeneous in terms of diagnostic criteria, criteria for relapse/recurrence, type of antidepressants and observation period. Almost all studies compared antidepressants with placebo, which may limit the transferability to clinical practice, because antidepressants should be accompanied by psychological treatment [8, 9, 11]. The evidence supporting the protective role of psychological treatment has been inconsistent and inconclusive [10], and even fewer studies have investigated combinations of treatment measures typically provided in primary care [10]. Therefore, knowledge about the effectiveness of the provided depression care in general practice for patients with recurrent depression is essential. This includes both pharmacological and psychological treatment measures, to prevent further relapse or recurrence.

To describe the course of healthcare provision over time, trajectory-based methods identifying distinctive groups of individual trajectories within a population are relevant [12]. Previous studies using trajectory-based methods have focused on antidepressant response trajectories to describe response patterns measuring depression symptom outcomes [13, 14] and relapse trajectories among those who either continued or discontinued antidepressant treatment [15]. Additionally, some studies have analysed depression symptom trajectories in primary care patients, to identify those who were at greater risk of symptom persistence or more severe course [16, 17]. However, little is known about treatment trajectories that include different treatment measures among patients with recurrent depression. Such knowledge can provide clearer guidance for clinicians, especially in the context of preventing recurrence.

In this study, we estimated treatment trajectories for patients within the first year of a recurrent depressive episode. Further, we aimed to explore whether the estimated trajectories were associated with a subsequent depressive episode within one year.

## Methods

### Settings

All inhabitants in Norway have equal access to public health care services and prescription drugs, including antidepressants, covered by the National Insurance Scheme. A national list- based system (the Regular GP scheme), ensures that all residents are entitled a regular GP.

GPs provide comprehensive care for a broad range of health issues, and act as gatekeepers to specialist health care. Publicly funded treatment in specialist health care by psychologist or psychiatrist requires a referral from the GP and is usually provided to patients with complex conditions, recurrent or severe depression.

### Design

We conducted a nationwide registry-based cohort study using data from “The Norwegian GP-DEP Study” (Project title: “The regular general practitioner scheme: integrated and equitable pathways of depression care, facilitating work participation”) [18]. The current study sample is drawn from the closed “GP-DEP” cohort and comprised all individuals ≥ 18 years with depressive episode(s) in general practice in 2008–2012 and a recurrent (index) episode in 2012. We examined the association between GP depression care trajectory and a subsequent depressive episode within one year.

### Data sources

Data from five national registries for the period 2008 through 2016 were linked at the individual patient level using the unique personal identity number (encrypted) assigned to all Norwegian residents. All data was stored and analysed at a safe server at the University of Bergen, Norway.

From the *Population Registry*, we obtained data regarding gender, year of birth and, eventually, year of death and emigration. From the *National Educational Database*, we received information on the highest completed educational level. The *Control and Reimbursement of Health Care Claims* (KUHR) database stores data on all fee-for-service claims from public primary care providers. KUHR provided information on all encounters with a GP during daytime with a recorded diagnosis of depression (P76) according to the International Classification of Primary Care, 2nd version (ICPC- 2), date of contact and reimbursement code(s) for diagnostic and therapeutic measures, as recorded by the GPs. Additionally, we used ICPC- 2 diagnostic codes from all GP contacts to estimate comorbidity. *The Norwegian Patient Registry* (NPR) comprises information on all patient contacts with public specialised health care. We obtained information on all contacts with a recorded diagnosis of depression according to the International Classification of Disease 10 th version (ICD- 10) in psychiatric wards, as inpatients or outpatients. *The Norwegian Prescription Database* (NorPD) contains information on all prescription drugs dispensed to individual patients treated in ambulatory care. For each prescription of an antidepressant drug, NorPD provided date of dispensing, generic drug information (Anatomical Therapeutic Chemical (ATC) code), and reimbursement code (drugs reimbursed by the Norwegian State for the treatment of depression).

### Definitions

In this study, we examined *depression care episode(s)*. A depressive episode *started* with a consultation in general practice with a depression diagnosis (P76) recorded in KUHR. An episode *ended* when there had been no contacts related to depression (P76) the subsequent 365 days after the last contact. This was defined as the *contact-free interval*. For all episodes, start- and end dates were identified. An index episode was defined as a depressive episode that started during 01.01.− 31.12.2012. Given that the length of index episodes varied, and that the study period ended on 31.12.2016, a one-year observation period to identify any subsequent depressive episode was defined as a 12-month period following the contact-free interval after the end of the index episode.

### Study population

**Fig. 1:**
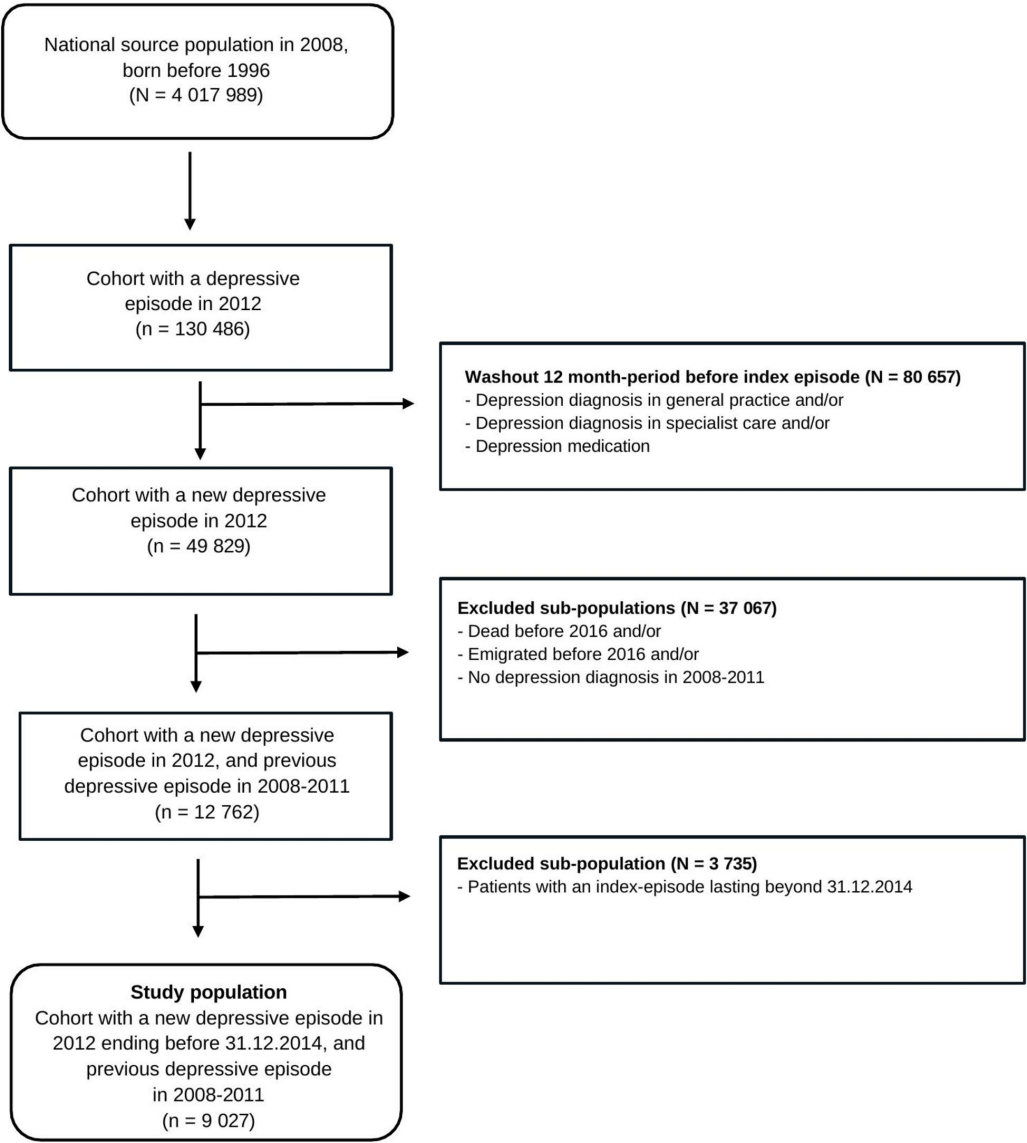
Flowchart illustrating the definition of the study population

The source population comprised all inhabitants of Norway born before 01.01.1996 and alive 01.01.2008 (4 017 989 individuals) (Fig. 1). First, we identified all individuals with a depressive episode in general practice (ICPC- 2 code P76 Depression in KUHR) in 2012 (*n* = 130 486). Second, to establish a cohort with a new depressive episode, we conducted: (i) washout of 80 657 individuals with a depression diagnosis in general practice (P76 in KUHR) and/or ii) specialist care (ICD- 10 codes F32, F33, F34 or F41.2 in NPR) and/or dispensed antidepressant drug (ATC code N06 A, reimbursed by the Norwegian State for depression treatment in NorPD) during the 12 months *prior to* the onset of the index episode*.* Third, to establish a cohort with recurrent depression course, we excluded patients without a previous depressive episode in 2008–2011 (*n* = 35 390), and those who died (*n* = 1 198) or emigrated (*n* = 479) during the study period. Finally, to allow observation time regarding any subsequent depressive episode until the end of the study on 31.12.2016, we excluded patients with index episodes lasting beyond 31.12.2014 (*n* = 3735). The final study population consisted of 9 027 patients.

### Explanatory variables

GP depression care trajectory was the main exposure variable. We estimated five different trajectories over *the course of 12 months from the onset of index depressive episode* (henceforth *index episode*), using group-based multi-trajectory modelling, as described under Statistical analysis. The trajectories were based on GP follow-up consultation(s), GP long consultation(s) and/or talking therapy, antidepressant drug treatment, and treatment in specialist mental healthcare (outpatient and/or inpatient care) for each month. The prerequisites for the reimbursement code for talking therapy were changed during the study period. Until July 2014, this code could not be combined with the reimbursement code for long consultation (> 20 min). Therefore, we combined these two reimbursement codes, both regarding treatment measure frequencies and when applied in trajectory models. The variables were recorded as binary (yes or no) for each month, except for follow-up consultation(s) that was summed up for each month.

### Outcome

Subsequent depressive episode was defined as a depressive episode (P76) registered in general practice within one year, which started at least 365 days (contact-free interval) after the end of the index episode. The outcome was binary (yes/no).

### Covariates

Gender was recorded as men and women. Patients’ age was categorised into six groups: 18–29, 30–39, 40–49, 50–59, 60–69 and 70 + years. Educational level was recoded from 11 levels into three categories: low [primary school (Grades 1–7) and lower secondary school (Grades 8–10)], medium (upper secondary school, Grades 11–13) and high (> 13 years, university, and higher education). Patients’ comorbidity was estimated, based on all GP-recorded diagnoses during 2009–2011. The diagnoses were derived from a list of common, chronic conditions established in general practice in Scotland by Barnett et al. [19] and adapted to ICPC- 2 codes used in general practice in Norway. Comorbidity was categorised as none, 1–2, or 3 + conditions.

### Statistical analyses

Descriptive statistics (frequencies with percentage) were used to examine the distribution of the covariates. The provision of GP depression care during the index episode was presented for those with and without a subsequent depressive episode. We presented median number of patients who received the various treatment measures with 25^th^-75^th^ percentile. Group-based multi-trajectory model (GBTM) was applied to identify latent subgroups of individuals following similar treatment trajectories based on depression treatment measures given each month during the index episode. GBTM is an application of finite mixture modelling, designed to identify groups with similar patterns over time [20], which distinguishes and identifies subgroups of treatment pathways in the study population. The GP consultation variable was used as Poisson distributed, while the other three depression care variables were binomially distributed. All variables were handled with a quadratic function over time. Models with two to seven groups were evaluated based on Bayesian information criteria (BIC), average posterior probabilities (APP) of assignment to trajectory groups, evaluation of distribution of individuals and clinical relevance of the trajectory groups (Additional file 1). The individuals were assigned to the trajectory group where their posterior probability of membership was highest [20]. Based on the best fitted treatment trajectory model, generalized linear model (GLM) was applied to investigate the association between treatment trajectories and subsequent depressive episode. The association was presented as relative risk (RR) with 95% confidence interval (CI). Crude estimates and estimates adjusted for patients’ age, gender, educational level, and comorbidity were calculated, but only the crude estimates were presented. Descriptive statistics (frequencies with percentage) were used to examine the distribution of demographic variables in the trajectory groups (Additional file 2). All analyses were performed using STATA/SE version 18.0 (Stata Statistical Software).

## Results

The study population consisted of 9 027 patients with recurrent depression course in general practice, mean age 44.6 (SD = 14.6) years, 63.9% were women. A total of 2 505 (27.8%) patients had a subsequent depressive episode (Table 1). Women, patients aged 40–49 years, those with medium education and those with one or two comorbid conditions made up the largest proportions. In terms of proportion of patients and number of times patients received a treatment, the provision of all treatment measures was marginally higher among those with a subsequent depressive episode compared to those without (Table 2).

**Table 1:** The study population (*N* = 9 027), by subsequent depressive episode, distributed by gender, age, education, and comorbidity

	**Study population**		**Subsequent depressive episode**			
			No, *n* = 6 522		Yes, *n* = 2 505	
	**n**	**%**	n	%	n	%
**Gender**						
Women	5 768	63.9	4 144	63.5	1 624	64.8
Men	3 259	36.1	2 378	36.5	881	35.2
**Age, years**						
18–29	1 732	19.2	1 302	20.0	430	17.2
30–39	2 056	22.8	1 459	22.4	597	23.8
40–49	2 247	24.9	1 588	24.3	659	26.3
50–59	1 673	18.5	1 182	18.1	491	19.6
60–69	884	9.8	672	10.3	212	8.5
70 +	435	4.8	319	4.9	116	4.6
**Educational level**^**a**^						
Low	3 021	33.9	2 204	34.2	817	33.1
Medium	3 650	41.0	2 623	40.8	1 027	41.5
High	2 238	25.1	1 609	25.0	629	25.4
*Missing*			86		32	
**Comorbidity**						
0	3 580	39.7	2 590	39.7	990	39.5
1–2	4 593	50.9	3 303	50.6	1 290	51.5
3 +	854	9.4	629	9.7	225	9.0

^a^Educational level: Low = primary school (grades 1–7) and lower secondary school (grades 8–10), or less; Medium = upper-secondary school; High = university and higher education

**Table 2:** Depression care measures^a^ provided during index episode, by subsequent depressive episode

	**Subsequent depressive episode**			
	**Yes, *n* = 2 505**		**No, *n* = 6 522**	
	**Patients who received treatment**	**Number of times patients received a treatment**^**b**^	**Patients who received treatment**	**Number of times patients received a treatment**^**b**^
**Depression care measures**^**a**^	**n (%)**	**median (25 th- 75 th percentile)**	**n (%)**	**Median (25 th- 75 th percentile)**
Follow-up consultation(s) with GP	1 648 (65.8)	4 (2–7)	3 929 (60.2)	3 (2–6)
Long consultation(s) and/or talking therapy with GP^c^	2 149 (85.8)	2 (1–4)	5 326 (81.7)	2 (1–3)
Antidepressant drug	638 (25.5)	2 (1–5)	1 624 (24.9)	2 (1–4)
Specialist health care contacts^d^	262 (10.5)	7 (2–14)	627 (9.6)	6 (2–13)

^a^Patients may have received more than one depression care measure

^b^Only those patients who received the depression care measure

^c^Including treatment given in index consultation

^d^Outpatient contacts and/or hospital admission

### Depression care trajectory groups and their treatment characteristics

**Fig. 2:**
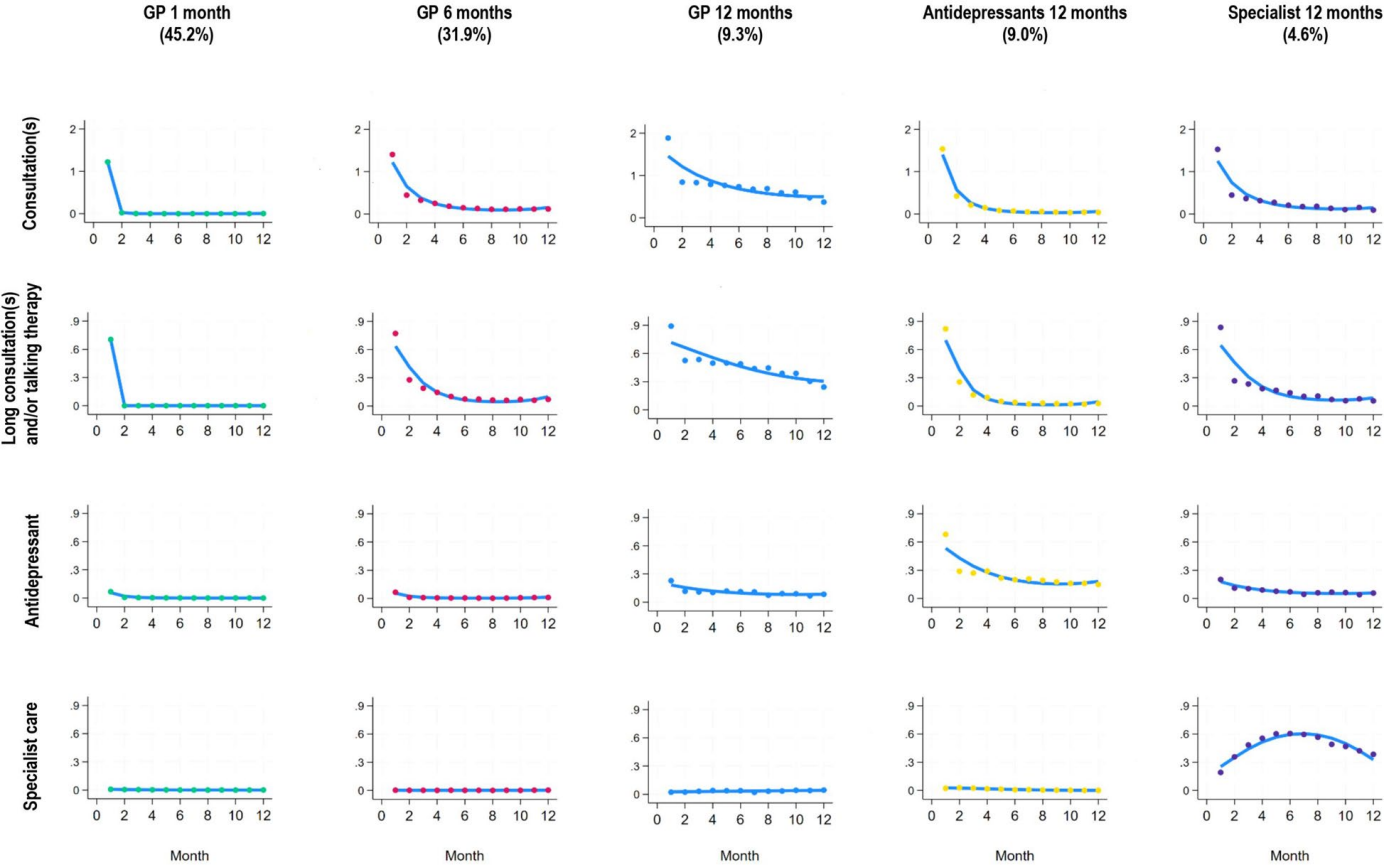
Treatment trajectories based on depression care measures provided during index episode

The group-based multi-trajectory modelling identified five subgroups of individuals who followed similar trajectories of depression care during the first year from the start of the index episode (Fig. 2).

The trajectory group *“GP 1 month” (GP care during the first month)* comprised 45.2% of the study population. This trajectory group was characterized by patients who received a GP consultation during the first month, among these 70% were long consultation(s) and/or talking therapy. The following 11 months, this group had no further registered depression treatment. Individuals in the trajectory group “*GP 6 months*” (31.9%) received GP care during six months. The patients had a GP consultation during the first month, among these 78% were long consultation(s) and/or talking therapy. Thereafter, the frequency of GP consultations decreased rapidly. The trajectory group”*GP 12 months*” (9.3%) consisted of patients who received GP care during 12 months. They had averagely two GP consultation during the first month, almost 90% were long consultation(s) and/or talking therapy. The following five months, 40–50% of the individuals were provided long consultation(s) and/or talking therapy, thereafter, slowly decreasing to around 30% last month. The first month, 22% collected medication, about 10% in the remaining 11 months. Individuals in the “*Antidepressants 12 months*” group (9.0%) differed from the other groups by consistently collecting medication throughout one year, with 70% collecting prescriptions in the first month and approximately 25% in the final months. They had a GP consultation the first month, among these 83% were long consultation and/or talking therapy the first month, about 10% month 4. The last trajectory group, “*Specialist 12 months*” (4.6%) was characterised by high degree of specialised mental healthcare throughout one year, 20% first month, increasing to 60% month 6, and thereafter decreasing to about 40% last month. The vast majority had a GP consultation in the first month, of which 80% had a long consultation and/or talking therapy, approximately 10–30% in the remaining period. The first month, 20% collected medication, about 11 to 4% in the remaining 11 months. In terms of patient characteristics, the younger, the well-educated and those with no or few comorbid conditions received follow-up with GP or specialist over a year to a greater extent than the older, the poorly educated and those with more comorbid conditions (Additional file 2).

### Depression care trajectories and subsequent depressive episode

The absolute risk of a subsequent depressive episode varied between 25.1% for the trajectory group “*GP 1 month*” and 35.8% for the “*GP 12 months*” group. Compared to *“GP 1 month”* (reference group), we found an increased risk of subsequent depression for all trajectories, except for *“Antidepressants 12 months*” (Table 3). Individuals in the “*GP 12 months*” group had the highest likelihood of a subsequent depressive episode (adj RR = 1.43, CI 1.29–1.59), followed by “*Specialist 12 months*” (adj RR = 1.26, CI 1.07–1.48) and “*GP 6 months*” (adj RR = 1.17, CI 1.08–1.27). Adjusting for patients’ gender, age, educational level, and comorbidity had no impact on the calculated estimates.

**Table 3:** Absolute risk and relative risk^a^ of subsequent depressive episode, by depression care trajectory groups

			**Subsequent depressive episode**	
**Depression care trajectory groups**	**Trajectory group** **n**	**Number and percentage of trajectory group** **n (%)**	**Crude RR (95% CI)**	**Adjusted**^**b**^** RR (95% CI)**
**1**. GP one month	4 080	1 059 (25.1)	1	1
**2.** GP six months	2 880	834 (29.3)	**1.17 (1.08–1.26)**	**1.17 (1.08–1.27)**
**3**. GP 12 months	839	295 (35.8)	**1.43 (1.29–1.59)**	**1.43 (1.28–1.59)**
**4.** Antidepressants 12 months	812	192 (26.0)	1.04 (0.91–1.18)	1.04 (0.93–1.19)
**5.** Specialist 12 months	416	131 (31.6)	**1.26 (1.08–1.46)**	**1.26 (1.07–1.48)**

^a^Results from generalized linear model (GLM) estimating relative risk (RR) with 95% confidence interval (CI)

^b^Adjustment for gender, age, educational level and comorbidity did not change the estimates

## Discussion

### Main findings

In a cohort of 9 027 adult patients with recurrent depressive episodes, we examined treatment trajectories and their association with a subsequent depressive episode within one year. Five treatment trajectory groups emerged: the “*GP 1 month”* group w*as characterised by patients receiving GP care during the first month.* “*GP 6 months” and “GP 12 months” group* consisted of patients who received GP care during six and 12 months respectively. The *“Antidepressants 12 months”* group differed from the other groups by collecting medication throughout one year, while the*”Specialist 12 months”* group was characterised by high degree of specialised mental healthcare throughout one year We found that one in four patients had a subsequent depressive episode. The risk of subsequent depression was lowest among individuals in the trajectory group”*GP 1 month” (*reference). Among those treated with antidepressants throughout one year (*”Antidepressants 12 months”)*, the likelihood of subsequent depression was not significantly different from the reference. In contrast, the trajectory groups receiving longer follow-up and treatment from GP and/or specialist (“*GP 6 months”,* “*GP 12 month”’, “Specialist 12 months”*) had a 17–43% higher likelihood.

### Interpretation of findings and comparison with existing literature

#### Recurrence rate of depressive episode

The 28% recurrence rate of depressive episode observed within one year in this study is consistent with other European studies from general practice. Recurrence rates varied between 28% over one year in Estonia [21], 34% over two years in Sweden [22] and 42% over seven years in Finland [23]. A study from Spain revealed a 40% recurrence rate within 1 to 1.5 years [24], while a study from the Netherlands reported recurrence rates of approximately 50% within one year after remission [25]. The variation in recurrence rates can be explained by differences in the definition of recurrence/relapse, study population, observation period, and depression care received [26, 27]. Differences in observation period and treatment measures makes it difficult to compare the rates directly. Nonetheless, recurrence rates did not increase much with a longer observation period, supporting that most recurrences occur early [2].

#### Treatment trajectories

Not surprisingly, individuals in the *“GP 1 month”* group (reference) had the lowest risk of recurrence. This is probably due to a low symptom burden and a self-limiting course, without the need for treatment and follow-up. Although several meta-analyses show robust evidence on the protective effect of antidepressants in preventing relapse and recurrence of depression [8, 9, 11], some trials have shown only a modest protective effect [15]. Therefore, our finding of a similar risk of recurrence in the *“Antidepressants 12 months”* and the *“GP 1 month”* group trajectory is of particular interest. Drug treatment is often recommended for patients with moderate to severe depression, and we therefore assume that patients in the “*Antidepressants 12 months”* group experienced more severe symptoms and thus had a higher risk of recurrence. NICE guidelines on depression management in adults recommend for individuals at risk of recurrence – defined as having had two or more episodes, residual symptoms or severe or prolonged episodes – to maintain medication for up to two years after remission [28]. Our findings support the protective effect of antidepressants among those with recurrent depression. But given the disparities in methodological approach, direct comparisons with other studies are challenging. Systematic reviews are mainly based on studies comparing antidepressant treatment with placebo controls, mainly conducted in specialist mental healthcare [8, 9, 11]. For instance, a systematic review and meta-analyses of controlled trials evaluating the effectiveness of various treatments on prevention of relapse and recurrence, revealed that patients taking antidepressants were twice as likely to avoid a recurrence compared to those taking placebo [10]. Further, the systematic review concluded that the results for psychosocial interventions were inconclusive and identified a lack of evaluation of combined treatment measures [10]. In contrast, the present study investigates various depression treatment measures provided within 12 months, as a part of a naturalistic follow-up and includes follow-up contacts with GP or specialist and non-pharmacological treatment.

The results of our study add new insights into the potentially protective effect of long-term antidepressant treatment for patients with recurrent depressive episodes. Guidelines recommend that treatment with antidepressants should last at least six months [28]. While long-term use can be beneficial in terms of remission, it may also pose a risk of adverse effects. A Scottish study documented that one in four long-term users of antidepressants had potential indications for deprescribing, related to increased fall risk, cardiovascular risks, and insomnia [29]. Therefore, it is important that clinicians assess the necessity of long-term use, especially among elderly patients and those with polypharmacy, who are at higher risk of adverse effects [29]. This requires clinicians assessing and discussing with their patients on the risk and benefits of long-term antidepressant use, ensuring informed decision-making.

The trajectory groups with GP- or specialist care throughout one year were most likely to have a subsequent depressive episode. Although we lacked information on severity, our findings may indicate that these trajectory groups comprised patients with more severe depression than the reference group, with a greater need for treatment and close follow-up. Studies have shown that severe symptoms in the index episode increased the likelihood of recurrence [30], supporting our interpretation of high level of treatment among those with severe burden of depressive symptoms. There is no reason to believe that long-term treatment itself can contribute to increased risk of subsequent depression episode(s). However, prolonged treatment may reflect treatment-resistant depressive disorder. Further, our finding that younger, well-educated or healthier patients received follow-up with GP or specialist over a year to a greater extent than older, poorly educated or multimorbid patients, indicates that other patient characteristics than the severity of the disease also may affect the GP's treatment strategy and thus the probability of belonging to a particular trajectory group. Variation in GP depression care across population groups has been demonstrated previously [18, 31].

To our knowledge, the present study is the first to investigate the association between treatment trajectories and the risk of subsequent depressive episode. A German primary care study assessed different depression symptom trajectories among patients receiving a collaborative care intervention [17]. In contrast to our study, the collaborative care included case management and behavioural activation, with one GP and one healthcare assistant assigned to the intervention group. The study identified two trajectories, “fast improvers” and “slow improvers”. Although collaborative care was effective in improving depression symptoms, the rate of improvement varied between patients. Patients who were “slow improvers” had higher symptom severity as baseline. If we assume that our trajectories of GP- or specialist care throughout one year have a higher baseline severity, these patients may be categorised as “slow improvers”, and therefore in need of prolonged or more intensive treatment.

In contrast to our finding of higher likelihood of recurrence among those with a high frequency of GP contact with long consultation(s)/talking therapy (“*GP 12 months”)*, there is evidence that patients who received cognitive behavioural therapy during an index episode of acute depression may have enduring effects in limiting risk of relapse or recurrence for up to two years after initial remission [32, 33]. Notably, this enduring effect was at least as efficacious as continuing antidepressant medication [33]. Another recently published study evaluating the effects of a team-based intervention in primary care, including cognitive behavioural therapy elements and case management supported by eHealth in patients with depression, indicated a reduction in the severity of depression symptoms [34]. However, this study did not specifically include those with a recurrent course, nor did they evaluate the lasting effect of the given intervention. In our study “talking therapy” refers to various types of psychological treatment, including supportive talk, counselling, and more structured psychotherapeutic methods such as cognitive-behavioural therapy. Therefore, our results are not comparable with studies examining specific psychological interventions, and may potentially explain differences in the findings.

Some treatment trajectories in the present study shed light on combinations of treatment modalities given. Despite our findings, the study design does not allow for conclusions about whether there is a causal relationship between the provided depression care and risk of recurrence. Notably, the absolute risk of recurrence ranged between 25–36%. While these figures may appear modest from a population perspective, they hold significant clinical relevance at the individual patient level, particularly if preventive interventions can effectively moderate the absolute risk.

### Strengths and limitations

A key strength of this study is the use of linked data from several national registers, providing a rich source of information, avoiding recall bias. The almost complete datasets are another strength; missing was limited to 1.3% (*n* = 118) on education with no expected impact on the results, due to small numbers. GP-registered depression diagnosis was defined as a GP consultation with ICPC- 2 code P76, after one-year washout. Differing coding behaviour can potentially challenge the internal validity. Nonetheless, this would apply to all GPs and cause non-differential misclassification and thus would not introduce bias in the results.

The definition of a subsequent depression episode with a contact-free interval (12 months), affects the identification of new depressive episode(s). We used the standardized approach proposed by Nielen et al. [35] to avoid under- and overestimation of depression episodes and to enhance both the consistency and comparability of our findings. Nevertheless, we acknowledge that patients seeking treatment for residual symptoms might be incorrectly classified as experiencing a new episode rather than a continuation of the previous one. This can lead to an overestimation of the incidence of subsequent depressive episode(s).

The lack of information on severity of depression is a limitation, as the ICPC system does not allow such grading. However, the inclusion of patients with a recurrent course of depression, i.e. at least two depressive episode(s) during 2008–2012, indicates a certain homogeneity in severity. 

NorPD contains data on dispensed medication; thus, the prevalence of antidepressants prescribed will be slightly underestimated in this study. Further, we do not know whether patients used the drugs they collected, i.e. non-compliance. To strengthen internal validity, we included antidepressants only reimbursed for depression, and not for other conditions, such as anxiety disorders. Antidepressants are usually prescribed and collected for three months at a time in Norway. Because we use antidepressants dispensed each month in the trajectory group modelling, the actual one-month prevalence of antidepressant treatment will probably be underestimated in this study.

For the purpose of our study, we included educational attainment as the only proxy for socioeconomic status. Educational level alone does not account for variation in wealth or income. Nevertheless, education accounts for differences in occupational social class, and to a large extent, variation in income [36]. Thus, educational level is a suitable proxy for socioeconomic status in this context.

In order to establish a large study population with recurrent depressive episode(s), and to allow observation time to identify subsequent depressive episode(s), we included data for the whole study period available in the GP-DEP project from 2008–2016.

Estimating healthcare multi-trajectories provides insight in complex pathways that include more than one treatment modality at a time or successively. Yet, due to the lack of information on depression severity, we don’t know whether the association between treatment trajectories and subsequent depression reflects the effect of the given treatment and/or the trajectory group individuals’ risk of recurrence due to severity.

Our findings can be transferred to countries that have similar organisation of primary health care, such as the Nordic countries, UK, and the Netherlands.

## Conclusions

Depression recurrence is common in primary care, highlighting the need for GPs and patients with a history of depression to develop a structured follow-up strategy. Patients with recurrent depressive episodes can benefit from long-term antidepressant treatment, as this seems to have a preventive effect on subsequent depression. As GPs often deliver various depression care measures simultaneously or successively, future studies should explore more complex care pathways with combined treatment measures. Further, the severity of the depressive episode should be examined, to strengthen our understanding of recurrence prevention and to guide GPs to deliver optimal care.

## Supplementary Information

Additional file 1. Model fit for group based multi-trajectories.

Additional file 2. Distribution of patient characteristics by trajectory groups.

## Data Availability

The data used in this study are provided by Statistics Norway, the Norwegian Directorate of Health, and the Norwegian Institute of Public Health, with restrictions only to be used under licence for researchers in the current study, thus, they are not publicly available. However, the registry data used in this study will be available from the authors upon reasonable request and with included permission from the Regional Ethical Committee for Medical and Health Research Ethics, Region West, Norwegian Data Protection Authority, Statistics Norway, the Norwegian Directorate of Health and the Norwegian Institute of Public Health.

## Abbreviations

ATC: Anatomical Therapeutic Chemical
CI: Confidence Interval
GBTM: Group-based multi-trajectory model
GLM: Generalized Linear Model
GP: General Practitioner
ICD: International Classification of Diseases
ICPC: International Classification of Primary Care
KUHR: Control and Reimbursement of Primary Health Care Claims Database
NICE: National Institute for Health and Care Excellence
NorPD: Norwegian Prescription Database
NPR: Norwegian Patient Registry
RR: Relative Risk
SD: Standard Deviation
WHO: World Health Organisation
