# Supplementary material for: Depression care trajectories and associations with subsequent depressive episode: a registry-based cohort study (The Norwegian GP-DEP study)
Source: BMC Prim Care. 2025 Apr 24;26:123. doi: 10.1186/s12875-025-02825-x (PMC12023545; doi:10.1186/s12875-025-02825-x)
Supplement: Supplementary file 1 — Additional file 1. Model fit for group based multi-trajectories. [file 12875_2025_2825_MOESM1_ESM.docx]

| **Additional file 1. Model fit**^1^ **for** **group based multi-trajectories** | | | | | |
| --- | --- | --- | --- | --- | --- |
|  |  |  |  |  |  |
| **Groups** | **BIC**^2^ | **ΔBIC** | **ΔBIC %** | **Group % size** | **Average posterior probability** |
| 2 | -108 468 |  |  | 70, 30 | 98, 96 |
| 3 | -104 599 | 3869 | 3.7 | 42, 44, 14 | 97, 97, 95 |
| 4 | -101 656 | 2 943 | 2.9 | 40, 5, 45, 10 | 97, 96, 94, 97 |
| 5 | -99 947 | 1 709 | 1.7 | 9, 32, 5, 45, 9 | 96, 95, 94, 91, 97 |
| 6 | -99 219 | 728 | 0.7 | 21,13, 5, 49, 8, 8 | 96, 88, 85, 96, 92, 96 |
| 7 | -98 661 | 558 | 0.6 | 7, 13, 6, 21, 43, 5, 4 | 96, 88, 86, 90, 92, 89, 96 |

^1^We used Bayesian information criterion (BIC), group size (%) and average posterior probability as model fit statistics for evaluation of the multi-trajectory groups. Compared to four groups, five groups had a lower BIC. The BIC value did not reach a minimum value, but the BIC value changed only minimally from five to six groups. The smallest group size (%) was similar for four-, five- and six-group trajectories. The average posterior probability was better for five groups rather than six groups. Finally, with regards to clinical relevance, compared to the five-group trajectory model, the six-group model contained two nearly identical treatment trajectories.

^2^Bayesian information criterion
