# Supplementary material for: Depression care trajectories and associations with subsequent depressive episode: a registry-based cohort study (The Norwegian GP-DEP study)
Source: BMC Prim Care. 2025 Apr 24;26:123. doi: 10.1186/s12875-025-02825-x (PMC12023545; doi:10.1186/s12875-025-02825-x)
Supplement: Supplementary file 2 — Additional file 2. Distribution of patient characteristics by trajectory groups. [file 12875_2025_2825_MOESM2_ESM.docx]

| **Additional file 2. Distribution of patient characteristics by trajectory groups** | | | | | |
| --- | --- | --- | --- | --- | --- |
|  | **Trajectory Group** | | | | |
|  | **GP 1 month** | **GP 6 months** | **GP 12 months** | **Antidepressants 12 months** | **Specialist 12 months** |
| **Patient characteristics** | **n (%)** | **n (%)** | **n (%)** | **n (%)** | **n (%)** |
|  |  |  |  |  |  |
| **Gender** |  |  |  |  |  |
| Women | 2 636 (62.8) | 1 858 (65.2) | 537 (65.3) | 465 (62.9) | 272 (65.5) |
| Men | 1 563 (37.2) | 993 (34.8) | 286 (34.7) | 274 (37.1) | 143 (34.5) |
|  |  |  |  |  |  |
| **Age, years** |  |  |  |  |  |
| 18-29 | 793 (18.9) | 503 (17.6) | 164 (19.9) | 153 (20.7) | 119 (28.7) |
| 30-39 | 933 (22.2) | 623 (21.9) | 203 (24.7) | 165 (22.3) | 132 (31.8) |
| 40-49 | 1 024 (24.4) | 741 (26.0) | 226 (27.5) | 161 (21.8) | 95 (22.9) |
| 50-59 | 765 (18.2) | 568 (19.9) | 155 (18.8) | 141 (19.1) | 44 (10.6) |
| 60-69 | 470 (11.2) | 266 (9.3) | 59 (7.2) | 70 (9.5) | 19 (4.6) |
| 70+ | 214 (5.1) | 150 (5.3) | 16 (1.9) | 49 (6.6) | 6 (1.4) |
|  |  |  |  |  |  |
| **Educational level^1^** |  |  |  |  |  |
| Low | 1 420 (34.2) | 988 (35.1) | 253 (31.2) | 238 (32.7) | 122 (29.7) |
| Medium | 1 707 (41.1) | 1 140 (40.6) | 328 (40.5) | 320 (44.0) | 155 (37.7) |
| High | 1 023 (24.7) | 683 (24.3) | 229 (28.3) | 169 (23.3) | 134 (32.6) |
|  |  |  |  |  |  |
| **Comorbid conditions** |  |  |  |  |  |
| 0 | 1 706 (40.9) | 1 007 (35.6) | 310 (38.0) | 301 (41.0) | 190 (46.1) |
| 1-2 | 2 092 (50.2) | 1 508 (53.3) | 430 (52.8) | 369 (50.2) | 194 (47.1) |
| 3+ | 314 (8.9) | 314 (11.1) | 75 (8.5) | 65 (8.8) | 28 (6.8) |

^1^Educational level: Low = primary school (grades 1–7) and lower secondary school (grades 8–10), or less; Medium = upper-secondary school; High = university and higher education
